# Supplementary material for: Graphene-based synthetic antiferromagnets and ferrimagnets
Source: Nat Commun. 2017 Sep 26;8:699. doi: 10.1038/s41467-017-00825-9 (PMC5615057; doi:10.1038/s41467-017-00825-9)
Supplement: Supplementary file 1 — Supplementary Information [file 41467_2017_825_MOESM1_ESM.pdf]

Supplementary Note 1 Projected density of states (pDOS) analysis

In order to compare the electronic structure of antiferro (AFM) and ferro (FM) magnetic configurations we show in Supplementary Fig. 1 the calculated spin resolved density of states (DOS) of Fe and Co atoms projected on their $d_{3z^2-r^2}$ and $d_{yz/xz}$ states. Two different setups for each magnetic configurations have been plotted: with and without graphene, (a,c) and (b,d) panels, respectively. When graphene is present, Co and Fe pDOS shape changes drastically compared to those structures where graphene is not present. This trend is more important for the Fe electronic structure where d–up states are peaked around -3 eV whilst for those corresponding to the complete structure the d states are broader, from -0.5 eV up to -3.5 eV. In addition, the magnetic moment per Fe atom (MM_{Fe}) follows this behavior since its value go from 2.69 μ_B with graphene, up to 3.27 μ_B without it, making clear that d–up band is more populated than d–down band that has moved over the Fermi energy. It is worth to mention that this behavior occurs in the same fashion for AFM and FM configurations as we observe in the bottom panels of Supplementary Fig. 1. This means that the positive J=E_{FM}-E_{AFM} energy difference between the FM and AFM configurations must have an additional ingredient to make AFM stronger than FM. To this end we observe that the presence of the additional peak in Fe and Co d–down states at -0.5 eV only in the AFM–$Gr_{AB}Fe_A$ configuration will emphasize the Fe–Co hybridization. It is easy to check that this peak does not appear when there is no graphene and it appears only in Co for the FM–$Gr_{AB}Fe_A$ configuration, decreasing dramatically the hybridization between Fe and Co.

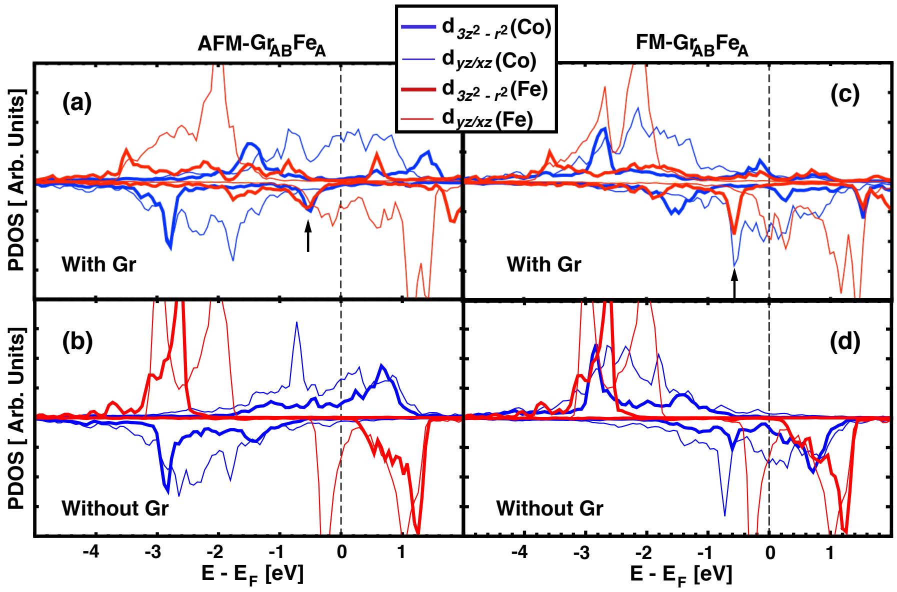

Supplementary Figure 1: **Projected density of states analysis.** AFM (a, b) and FM (c, d) pDOS for the optimized $Gr_{AB}Fe_A$ configuration. Thick solid colored lines show Fe/Co–$d_{3z^2-r^2}$ orbitals whilst light ones depict Co/Fe–$d_{yz/xz}$ orbitals. In addition, (a,c) panels show how the system behaves when Gr is present and (b,d) after remove it. The Black arrows depict the position of the characteristic peak of some structures.

Supplementary Fig. 2 shows the pDOS of Fe, $C_{1/2}$ and Co of $Gr_{AB}Fe_{(A,B,C)}$ configurations projected on their $d_{3z^2-r^2}$, $d_{yz/xz}$ and p_z states. The filled curve corresponds to the C atoms that

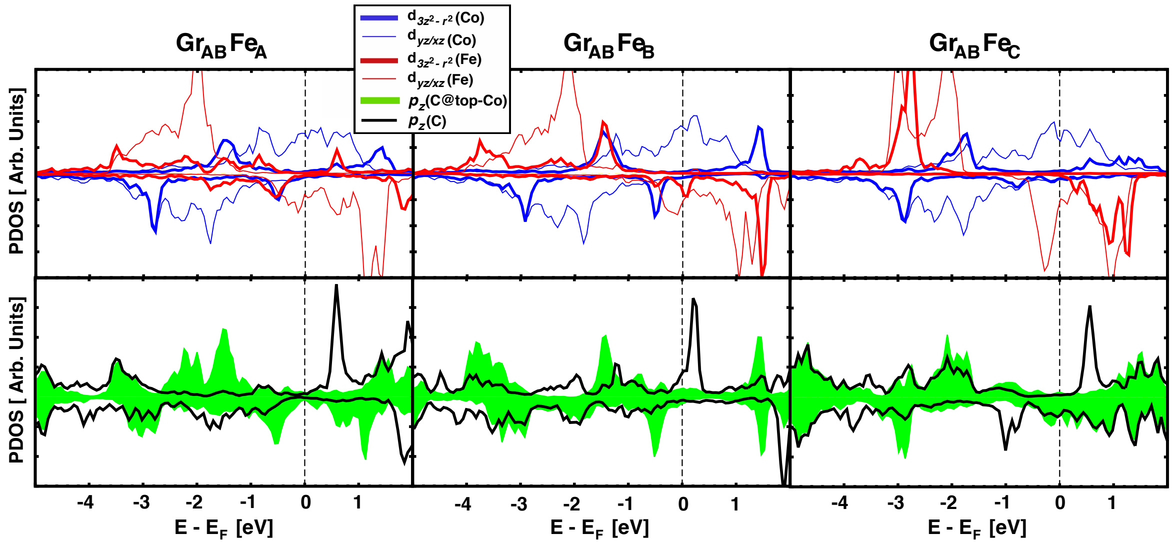

Supplementary Figure 2: **Selfconsistent antiferromagnetic projected DOS for the optimized $Gr_{AB}Fe_{(A,B,C)}$ configurations.** The upper panel follows the same color type as in the manuscript, i.e., thick (thin) red solid lines for Fe–$d_{3z^2-r^2}$ ($d_{yz/xz}$) and thick (thin) blue lines for Co–$d_{3z^2-r^2}$ ($d_{yz/xz}$). The bottom panes shows the pDOS of each carbon atom onto their p_z states having filled curves those C's that are @top–Co.

are on top of the Co whilst the black solid line shows the other C atom that can be either below the Fe or not, such as for $Gr_{AB}Fe_C$, where Fe is in the center of the graphene ring. The influence of graphene on the Fe–Co hybridization due to the presence of the p_z–down peak at -0.5 eV in $Gr_{AB}Fe_{(A,B)}$ becomes clear from the figures. When Fe is at the C site, this peak disappears. In fact, the exchange magnetic interaction J for $Gr_{AB}Fe_C$ is much weaker than in $Fe_{A,B}$ confirming that the presence of this peak supports the hybridization between Fe and Co and hence their strong AFM coupling.

	$\mathbf{Gr_{AB}}$			$\mathbf{Gr_{BC}}$			$\mathbf{Gr_{AC}}$		
	$\mathbf{Fe_A}$	$\mathbf{Fe_B}$	$\mathbf{Fe_C}$	$\mathbf{Fe_A}$	$\mathbf{Fe_B}$	$\mathbf{Fe_C}$	$\mathbf{Fe_A}$	$\mathbf{Fe_B}$	$\mathbf{Fe_C}$
$\mathbf{z}_{\text{Fe–C}}$	2.02	2.21	2.97	3.02	2.22	2.04	2.03	2.98	2.03
$\mathbf{z}_{\text{C–Co}}$	2.11	2.09	2.06	2.03	2.08	2.10	3.10	3.14	3.10
$\delta\mathbf{z}_{\text{C–C}}$	0.27	0.28	0.02	0.02	0.26	0.27	0.01	0.00	0.01
E_{ads}	1.66	1.62	0.34	0.33	1.40	1.52	1.12	0.26	1.13
J [mJ/m^2]	277	106	15.2	21.8	116.5	246	17.5	-8.7	17.5

Supplementary Table 1: **Atomic heights for the different Fe–graphene adsorption sites** Height between iron and one C atom of the Gr ML, $z_{\text{Fe–C}}$, carbon–cobalt out–of–plane atomic distance, $z_{\text{C–Co}}$ and roughness of carbon atoms within the Gr ML, $\delta_{\text{C–C}}$ of $\text{Gr}_{\text{AB/BC/AC}}\text{Fe}_{\text{A/B/C}}$ geometric configurations, rows 1, 2 and 3, respectively. The fourth row depicts the adsorption energy in meV and the last the exchange magnetic interaction, J, as defined in the main text.

Supplementary Note 2   k–points sampling test

As it was pointed out along the manuscript, the MAE values are of order of a few mJ m^{-2}. Consequently, the calculation of the self–consistent total energies involved have to be sufficiently accurate. We performed convergence tests for several relevant DFT parameters resulting in sufficient accuracy for the quantities under study. One of the most important quantity is the k–points sampling shown in Supplementary Fig. 3. In this figure is depicted the MAE convergence for the $Gr_{AB}Fe_A$ configuration with increasing k–points. The dashed black lines indicate our required accuracy of 0.2 mJ m^{-2}. We observe that in the range from 961 k–points (indicated in red in the figure) to 1400 all calculated values lie within the required tolerance. Consequently we decided to use 961 k–points to perform the calculation of the MAE for all the configurations.

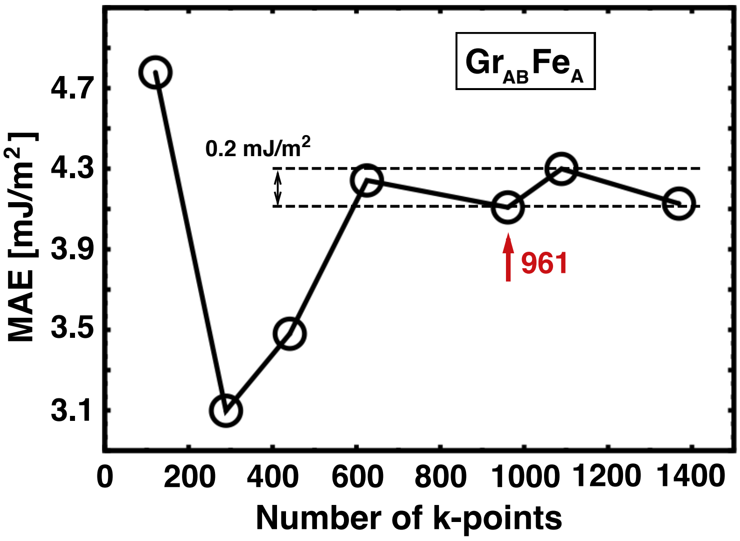

Supplementary Figure 3: **Magnetocrystalline anisotropy of the optimized $Gr_{AB}Fe_A$ configuration as a function of the number of the k–points.** The interval between the two dashed lines shows the magnetic anisotropy dispersion for the higher k–points values. The red arrow represents the number of k–points chosen in order to have accurate MAE values.

Supplementary Note 3 Sample preparation, Co intercalation morphology and Fe film morphology

Sample preparation has been done in situ with standard surface-science techniques in the BOREAS preparation chamber in a base pressure of $1 - 2 \times 10^{-9}$mbar. The Ir(111) single crystal (MaTecK) clean surface has been prepared by repeated Ar^{+} ions sputtering at 2-2.5 keV energy followed by $10'$ annealing at T=1200 K obtained on a electron borbament heating-station (FerroVac) followed by a $5'$ oxygen exposure at 8×10^{-7}mbar pressure at T=700 K, necessary to remove the excess carbon, and finally followed by a flashing to 1300 K in order to desorb the adsorbed oxygen from the surface. The clean sample was then checked by LEED in order to ensure the presence of the p(1×1) hexagonal pattern in Supplementary Fig.4, and the absence of any reconstruction due to oxygen or carbide adsorbed on the surface. The sample cleanness was also checked by AES for presence of oxygen and/or carbon.

The graphene layer has been prepared by chemical vapor deposition (CVD) of ethilene gas on the clean Ir(111) single crystal. The 2-2.5 keV Ar^{+} sputtered Ir(111) single crystal has been annealed at T=1200 K for $10'$ then, without letting the crystal to cooldown, it has been heated to T=1500 K while keeping the pressure in the $1 - 4 \times 10^{-8}$mbar range and subsequently exposed for $10'$ to a 1.2×10^{-6}mbar pressure of ethylene (C_2H_4) gas dosed via a leak valve in the preparation chamber. The sample was then let to cool down by removing the current supply to the heating station filament. The graphene layer was then checked on the sample at T$\simeq$RT via a mini-LEED (OCI Vacuum eng.). Gr/Ir(111) sample good quality was assessed by the sharpness of the LEED spots and by the visibility of at least two orders of Moiré pattern as reported in Supplementary Fig.4b). The Moiré pattern is typical of the Gr/Ir(111) surface and is related to the to the lattice mismatch and relative rotation of the graphene hexagonal mesh with respect to the Ir(111) hexagonal crystal lattice; the presence of well defined spots and several orders of Moiré can be taken as a good indication of the formation of large monodomain graphene covering a macroscopic area of the Ir(111) surface[1].

The Cobalt layer deposition was done by an e-beam evaporator in the sample preparation chamber. The Co evaporation rate was checked with a quartz microbalance prior evaporation and the Co coverage was subsequently estimated by the Co $L_{2,3}$ XAS edge jump to background ratio (see section Supplementary Note 5). The intercalation of Co is done by thermal annealing of the sample in a temperature range of T=500-700K. After the intercalation of 1ML of Co the Moiré pattern is preserved as previously reported [2, 3] although on the intercalated sample we were not able to image the same number of Moiré diffraction orders as reported for the Gr/Ir(111) Supplementary Fig.4c). The full Cobalt intercalation has been proved by exposing the intercalated sample to a partial pressure of oxygen in the sample preparation chamber, as discussed in section Supplementary Note 9.

The Fe overlayer morphology has been investigated by means of LEED diffraction pattern spot-width analysis [4]. The LEED pattern was calibrated to the Ir(111) reciprocal lattice size in order to scale the spot width in reciprocal lattice units (r.l.u.). The width of the specular diffraction spot, as obtained by a simple Gaussian peak fit, can be directly compared to the inverse average width of the Fe islands [4]. Due to the presence of the surface-backscattered visible light of the electron gun we performed the width analysis on the (1,0) diffraction spot. The results of the fitting reported in Supplementary Fig.5 are in agreement with an Fe average islads size of 6-8nm at a coverage of 1 ML.

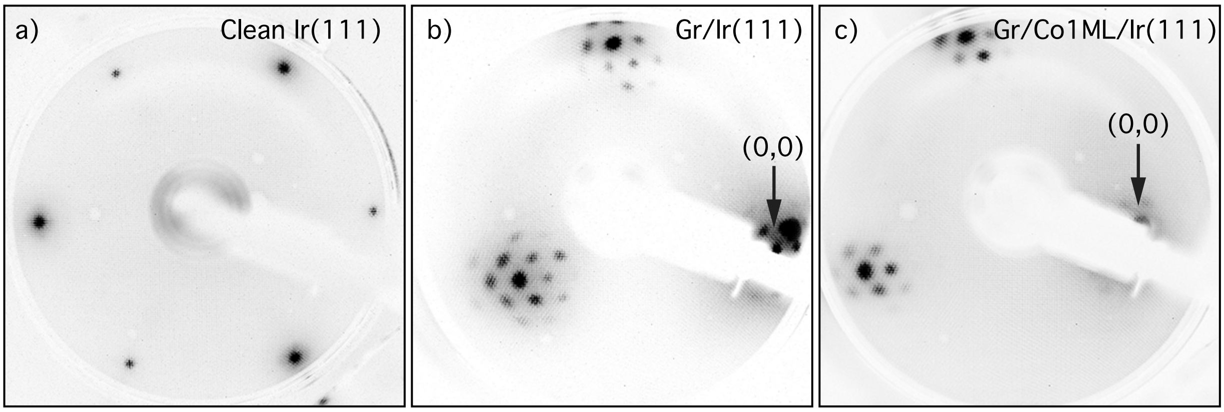

Supplementary Figure 4: **LEED patterns for different configurations of the system.** a) clean Ir(111) LEED pattern collected at 77eV of primary electron energy, showing the p(1×1) hexagonal pattern; b) Gr/Ir(111) LEED pattern collected at 44 eV of primary energy, on a slightly rotated on-axis ($\sim$20°) sample surface as to allow the first diffraction order enter the fluorescent screen of the mini-LEED (the (0,0) specular spot is identified by an arrow); c) Gr/Co[1 ML]/Ir(111) collected at 44 eV of primary energy in a comparable rotated angle as to panel b).

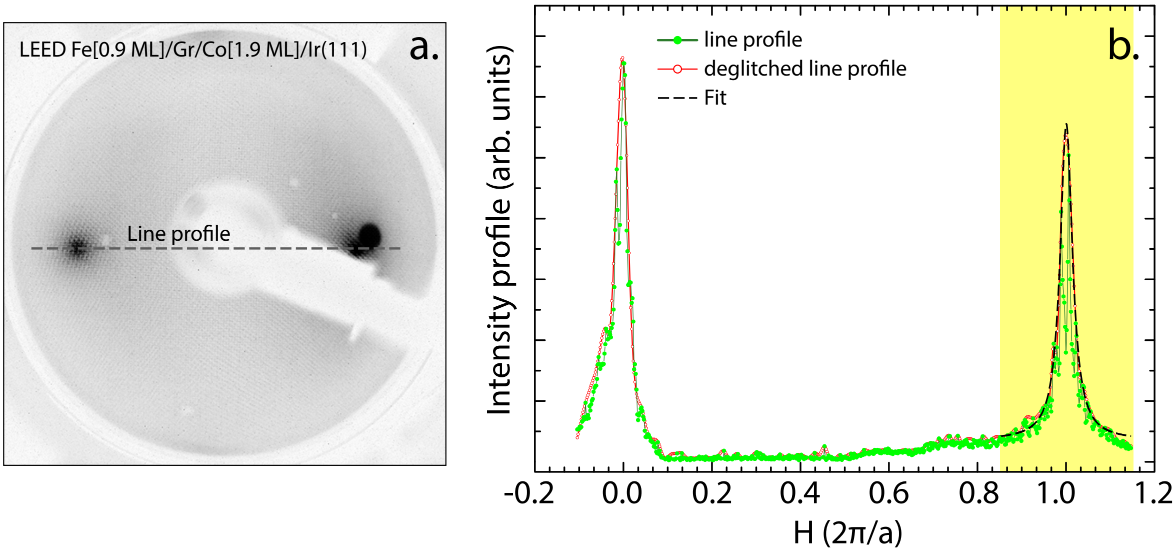

Supplementary Figure 5: **LEED intensity profile.** (a) LEED pattern collected at 46 eV primary energy for a Fe[0.9 ML]/Gr/Co[1.9 ML]/Ir(111) at room temperature; superimposed to the image is shown the line profile that gives the intensity profile shown in the right panel. (b) intensity line profile across LEED diffraction spots (green line/points) and fit (gaussian+lorentian, black dashed line) to the (0,1) diffraction spot used to estimate the average Fe island size at this coverage.

Supplementary Note 4 High resolution SEM images of bare and Co-intercalated Gr/Ir(111)

In Supplementary Fig.6 we report high-resolution Scanning Electron Microscope (SEM) images to provide further evidence, together with the LEED patterns, of the structural quality and morphology of our graphene and Co–intercalated graphene films on Ir(111). The images were obtained using the FEI HRSEM Magellan 400L instrument of the electron microscopy service at CIN2 laboratory, operated at a 1kV electron voltage for enhanced surface sensitivity while preserving high spatial resolution. In order to compare the bare Gr/Ir(111) and the Gr/Co/Ir(111) surfaces we have prepared a sample that present three different regions, as shown in Supplementary Fig.6(a), namely: one that corresponds to a bare Gr/Ir(111) interface, the second with a Co–intercalated graphene layer and the third region that is the boundary region between the Co–intercalated and the bare graphene one.

The SEM images of Supplementary Fig.6(b,c) collected in the bare graphene region at wide and medium scale respectively, show the smooth morphology of the graphene layer. In supplementary Fig.6(c), the typical graphene wrinkles are clearly seen together with essentially flat and free–of–defects areas at a $\sim$ 1µm scale, representing a very high (90% or higher) fraction of the surface coverage. A small percentage of darker patches is seen, and it might be interpreted as second– or multi–layer graphene islands. This findings agree with the self-limited single layer growth of graphene by CVD on the Ir(111) surface[5, 6]. We report in Supplementary Fig.6(d) a SEM image at medium scale collected in the region corresponding to Gr/Co/Ir(111). The image shows a flat morphology, with less–visible graphene wrinkles with respect to the bare graphene on Ir(111).

The image collected at the boundary between Co intercalated region and bare Gr/Ir(111) in Supplementary Fig.6(e) shows that the Co intercalation, seen in a darker color with respect to bare Gr/Ir(111), occurs principally at the wrinkles of the clean graphene layer in agreement with previous reports [7]. The progressive completion of a complete Cobalt layer do not produce any visible damage to the graphene layer which appears flat and homogeneous. The graphene wrinkles on the Co intercalated zone show a much weaker contrast with respect to the bare Gr/Ir(111) although some of them can be seen and have been marked by arrows in Supplementary Fig.6(e).

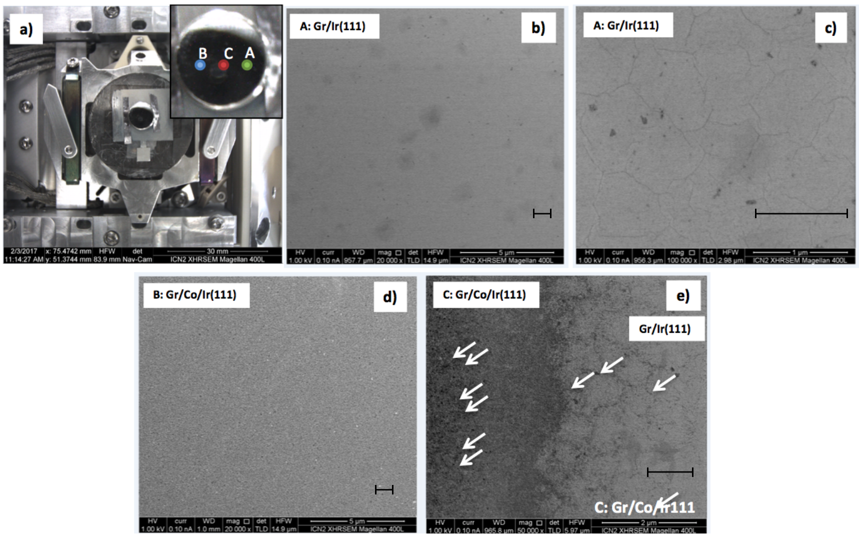

Supplementary Figure 6: **High resolution SEM imaging of a Co-intercalated Gr/Ir(111) sample.** (a) picture of the Ir(111) single crystal in the SEM setup, inset showing the 3 different zones of the sample (corresponding to A: bare Gr/Ir(111); B: Gr/Co/Ir(111); C: boundary region); (b,c) SEM images acquired in zone A, scale bar 1µm; d) SEM image acquired in zone B, scale bar 1µm; e) SEM image acquired at the boundary region C, scale bar 1µm. The white arrows indicate the most prominent wrinkles/ripples through which Co intercalation takes place.

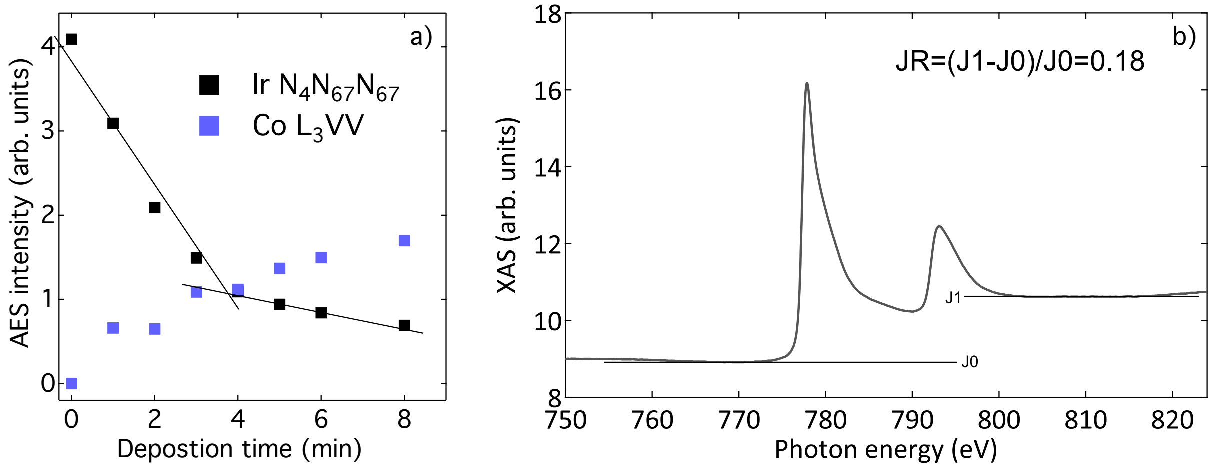

Supplementary Figure 7: **Coverage determination by AES and XAS.** a) AES peak-to-peal intensity of the Ir $N_4N_{67}N_{67}$ at 172 eV and Co L_3VV at 773 eV measured at different Co deposition time using the BOREAS mini-LEED AES setup. The change in slope of the substrate signal can be associated to the formation of 1ML of Co in layer-by-layer growth mechanism. b) Co $\mathrm{L}_{2,3}$ XAS spectrum corresponding to the AES slope change coverage point, and thus to a monolayer of Cobalt. The J_{R} value and the corresponding definition is reported in the inset.

Supplementary Note 5 Estimation of ML coverage by AES and XAS measurements

In order to calibrate the nominal coverage obtained by the quartz microbalance deposition rate monitor we performed an Auger electron spectroscopy (AES) study of the deposition of Co on bear Ir(111). The evolution of the AES peak intensities of the Ir $N_4N_{67}N_{67}$ at 172 eV and Co L_3VV at 773 eV (peak-to-peak) as a function of deposition time at constant deposition rate is reported in Supplementary Fig.7. The substrate AES line intensity as a function of deposition time shows a marked kink at about 4.5 min indicating the completion of the first Co layer. The subsequent slope change at higer deposition time is taken as an indication of the layer-by-layer growth in a Franck–van der Merwe mechanism.[8]

The sample obtained with an AES-calibrated one-monolayer Co coverage on bare Iridium was taken as a calibration for the XAS edge-jump to background ratio J_{R} in order to be able to estimate the coverage of the following preparation by XAS measurements. This method already described elsewhere[9] offers a fast and reliable way to determine the surface coverage below the XAS-jump saturation value that normally is attained above 10-12MLs. We obtain that a one monolayer coverage of Co/Ir(111) corresponds to an edge jump to background ratio of J_{R}=0.18±3.

Supplementary Note 6 Cobalt coverage dependent magnetic properties of Gr/Co/Ir(111)

In supplementary fig.8(a-d) we report the evolution of the hysteresis loop for Gr/Co/Ir(111) at increasing Co coverage. Below the ML completion small or no magnetic remanence is observed; at 1 ML coverage we observe a PMA hysteresis loop with 0.72% magnetic remanence and 0.4T coercivity. Further increasing the Co coverage to about 2.0 ML gives an almost perfect square-shaped loop with magnetic remanence of 92% and slightly lower coercivity of 0.23T indicating an almost single magnetic domain formation with high PMA.

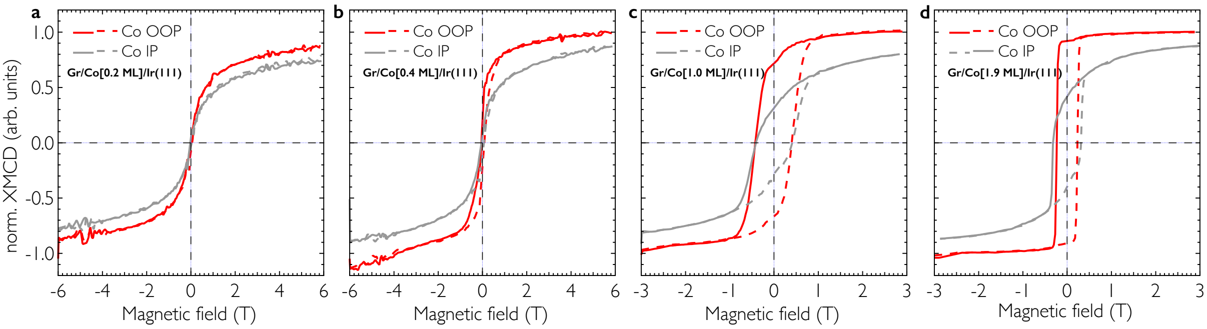

Supplementary Figure 8: **Thickness evolution of magnetization loops.** Evolution of the Co XMCD hysteresis loops, normalized to the saturation value, for different Co layer coverage (a) 0.2, (b) 0.4, (c) 1.0 and (d) 1.9 ML of Co.

Supplementary Note 7 Higher Fe coverage data

The AF coupling between Co and Fe is fairly preserved at various Fe coverages up to comparable Fe and Co thicknesses, as reported in the main text. Here we report further data completing the scenario of a Fe coverage larger than the Co one. Supplementary Fig.9a) shows XAS and XMCD spectra collected at remanent state for a Fe coverage equal to 1/3 of the Co one that is corresponding to 1.8$\pm$1 ML. The XMCD spectra indicates an antiparallel orientation of Fe and Co layers, evidencing their AF coupling. The situation for a Fe coverage corresponding to 2 times the Co one is depicted in Supplementary Fig.9b). In this case, both the Fe and Co remanent state XMCD show an inverted sign with respect to the previous case as well as with each other. We note that the Co remanent moment is reduced with respect to the previous case, indicated by a lower XMCD signal. The element specific hysteresis loops of the Co and Fe layers reported in Supplementary Fig.9c) further evidence the reduction of the Co moment, but most notably demonstrate that while a AF coupling is still present at zero field, now the Fe magnetization has become the dominant one remaining along the direction of the previously applied magnetic field and driving an inversion of the Co magnetization. It is also observed that the Co loop looses the square hard-magnet shape onto a more rounded loop. This behaviour could be related to domain formation on the Co layer induced by the AF coupling to the Fe, however this or other hypothesis remain to be further investigated.

Supplementary Note 8 AF coupling in the Co/Gr/Co/Ir(111) system

We performed an experiment on a Co layer deposited on top of a graphene-intercalated by Co 1.6 ML in order to investigate the nature of the coupling between homogeneous material interfaced to a graphene sheet. In Supplementary Fig.10 we report the out-of-plane room-temperature hysteresis loops acquired on a Gr/Co[1.6ML]/Ir(111) sample and subsequently on the Co[0.8ML]/Gr/Co[1.6ML]/Ir(111) sample realized depositing half the amount of cobalt that has been intercalated. In this case the chemical sensitivity of the XMCD does not help to separate the contribution of the substrate from the overlayer, however it is clearly visible that the XMCD signal (normalized to the maximum of the absorption edge to take into account the different amount of cobalt in the two samples) shows a reduced magnetic moment at B=0T and moreover a linear background superimposed to the squared loop of Gr/Co/Ir(111). These observations are suggestive of the presence of AF coupling on a Co/Gr/Co interface.

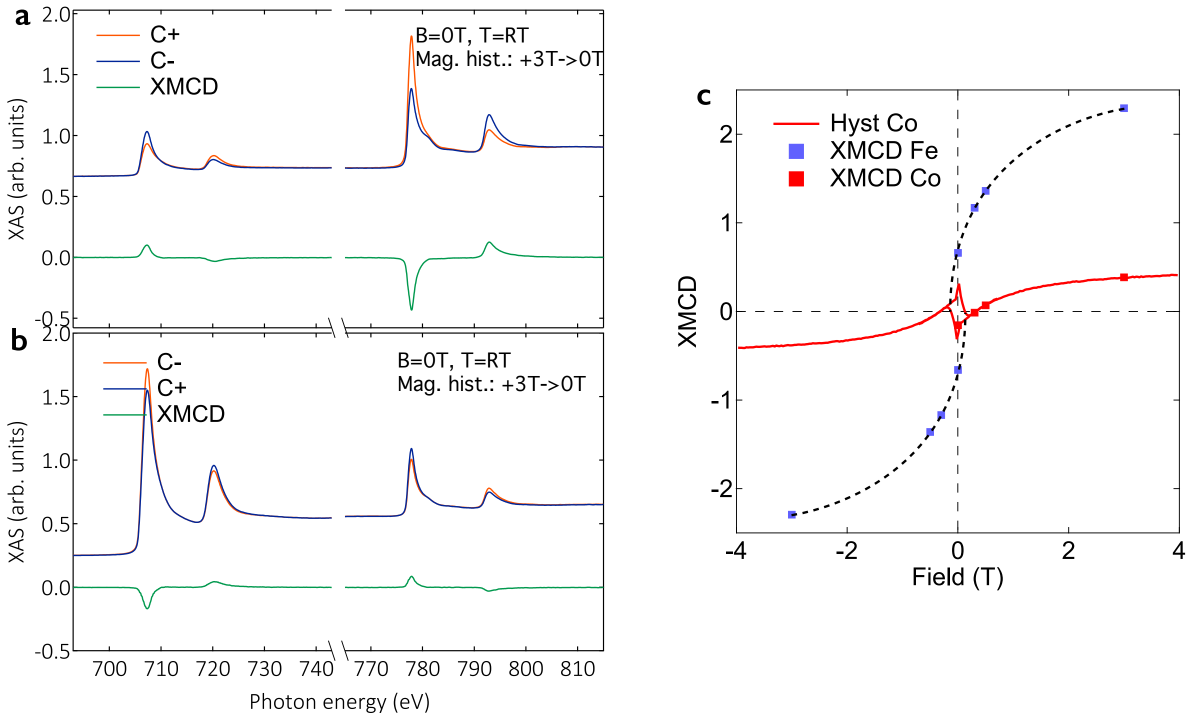

Supplementary Figure 9: **Reversed magnetization roles between Fe coverages lower and higher than the Co one.** Panels a),b) show Fe and Co $L_{2,3}$ XAS/XMCD collected under normal beam incidence at remanence, i.e. a field B=0T after having magnetized the sample with a positive field of B=3T, demonstrating a reversed role of the Co and Fe magnetizations when the Fe coverage is lower (a) and higher (b) than the Co coverage; (c) partial hysteresis loop collected on the high Fe coverage sample, showing the evolution of Co and Fe magnetizations as a function of field.

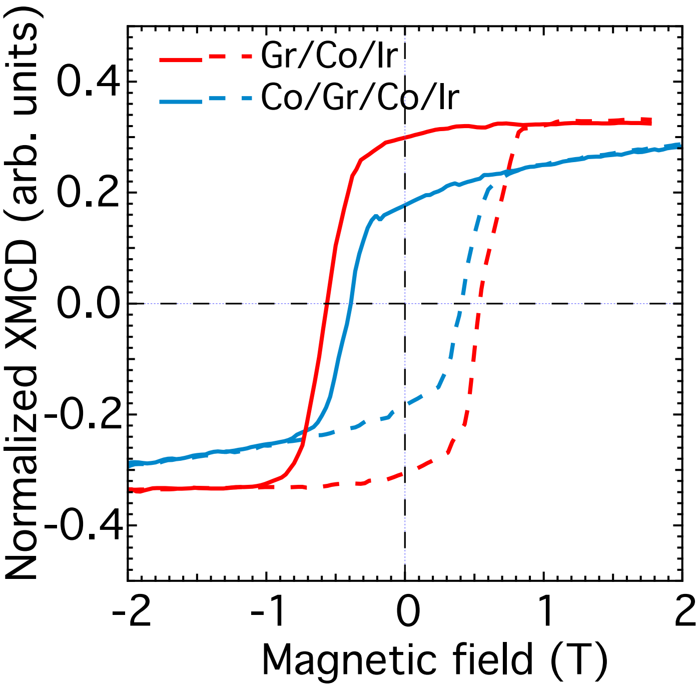

Supplementary Figure 10: **Magnetization loops in Gr/Co/Ir and Co/Gr/Gr/Ir samples.** Hysteresis loop collected along the easy axis (out-of-plane) for a Gr/Co[1.6ML]/Ir(111) sample (red lines) and for a Co[0.8ML]/Gr/Co[1.6ML]/Ir(111) (blue lines). The XMCD signal has been normalized to the maximum of the L_3 absorption edge in order to compare the loops with different amount of material.

Supplementary Note 9 Graphene-protected Co layer

The complete intercalation of the Co film below the Graphene sheet can be proved taking advantage of the passivating properties against oxidation of the Graphene layer.[10] In Supplementary Fig.11 we report the evolution of the Co L_3 edge of a pristine Gr/Co[1.9 ML]/Ir(111) (red curve) and after a 5 min exposure to a partial pressure of 1.0×10^{-6} mbar of molecular O_2 (green curve). The two spectra do not show any sign of Co-oxide formation indicating that: i) the graphene layer offers an effective barrier against oxidation, as already reported [10]; ii) the graphene coverage of the surface is complete so that we cannot observe sign of non graphene-covered Co atoms. As a comparison in 11 (black curve) we report the Co L_3 spectrum for a Co[1.9 ML]/Gr/Ir(111) sample exposed for 5 min to a partial pressure of 1.0×10^{-6} mbar of molecular O_2. In this case there are clear signs of divalent Co-Oxide formation as evidenced by the tick–marked peaks in the absortpion spectrum.

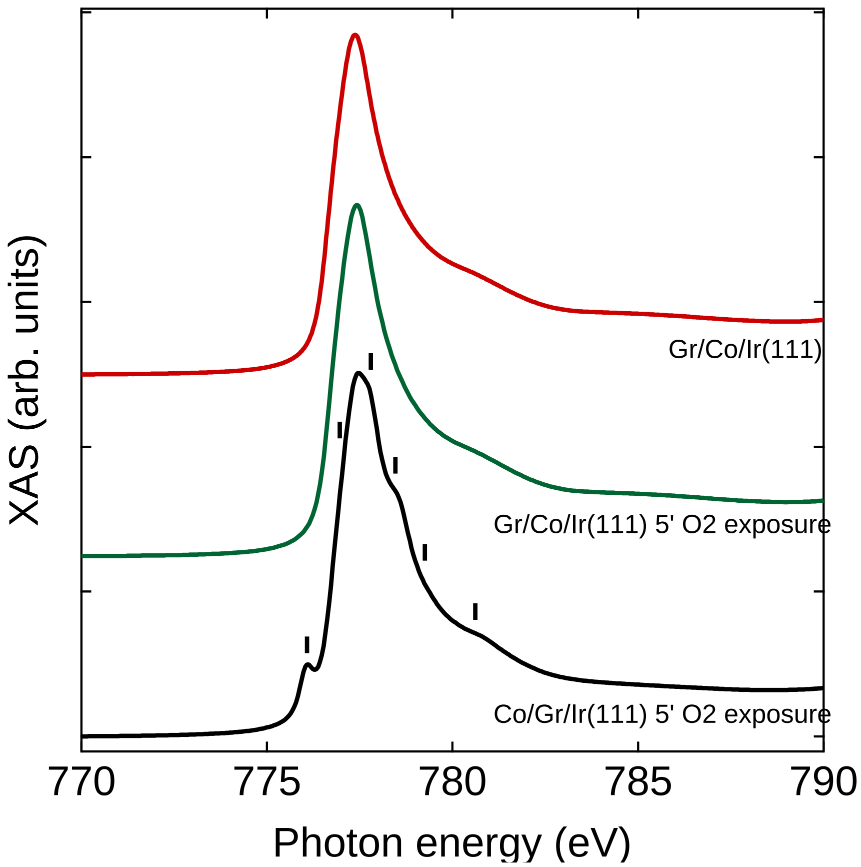

Supplementary Figure 11: **Verification of the completed intercalation by oxygen exposure.** Graphene-protected Co layer. From top to bottom: evolution of the Co L_3 absorption edge for a pristine Gr/Co[1.9 ML]/Ir(111) red curve; after 5 min 1.0×10^{-6} mbar of molecular O_2 exposure green curve; comparison with a non-intercalated Co[1.9 ML]/Gr/Ir(111) after 5 min 1.0×10^{-6} mbar of molecular O_2 exposure showing definite signs of CoO formation as evinced by the multiplet features tick–marked in the spectrum.

Supplementary References

[1] Hattab, H. *et al.* Growth temperature dependent graphene alignment on Ir(111). *Applied Physics Letters* **98**, 141903 (2011).

[2] Decker, R. *et al.* Atomic-scale magnetism of cobalt-intercalated graphene. *Physical Review B* **87**, 041403 (2013).

[3] Pacilé, D. *et al.* Electronic structure of graphene/Co interfaces. *Physical Review B* **90**, 195446 (2014).

[4] Henzler, M. Growth of epitaxial monolayers. *Surface Science* **357-358**, 809–819 (1996).

[5] Koh, S., Saito, Y., Kodama, H. & Sawabe, A. Epitaxial growth and electrochemical transfer of graphene on Ir (111)/α-Al_2O_3 (0001) substrates. *Applied Physics Letters* **109**, 023105 (2016).

[6] Coraux, J. *et al.* Growth of graphene on ir (111). *New Journal of Physics* **11**, 023006 (2009).

[7] Vlaic, S. *et al.* Cobalt intercalation at the graphene/iridium(111) interface: Influence of rotational domains, wrinkles, and atomic steps. *Applied Physics Letters* **104**, 101602 (2014).

[8] Biberian, J. & Somorjai, G. On the determination of monolayer coverage by Auger electron spectroscopy. Application to carbon on platinum. *Applications of Surface Science* **2**, 352–358 (1979).

[9] Arvanitis, D. *et al.* Experimental determination of orbital and spin moments from MCXD on 3d metal overlayers. In *Spin–Orbit-Influenced Spectroscopies of Magnetic Solids*, 466, 145–157 (Springer Berlin Heidelberg, 1996).

[10] Dedkov, Y. S., Fonin, M., Rüdiger, U. & Laubschat, C. Graphene-protected iron layer on Ni(111). *Applied Physics Letters* **93**, 022509 (2008).
